# Supplementary material for: Analysis of the Expression and Subcellular Distribution of eEF1A1 and eEF1A2 mRNAs during Neurodevelopment
Source: Cells. 2022 Jun 9;11(12):1877. doi: 10.3390/cells11121877 (PMC9220863; doi:10.3390/cells11121877)
Supplement: Supplementary file 1 [file cells-11-01877-s001.zip › cells-1746451-supplementary.pdf]

Supplementary material: Supplementary Figure S1.

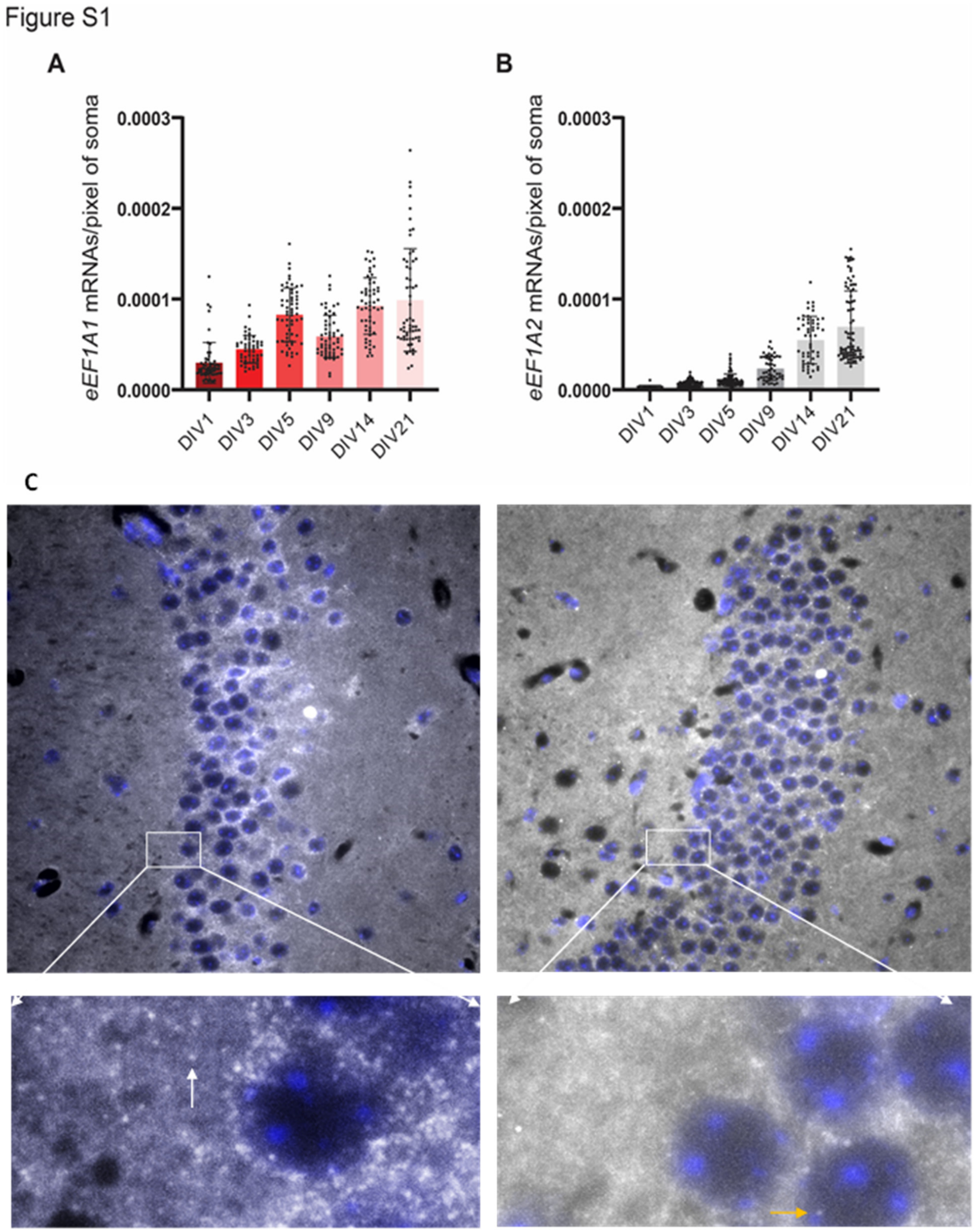

D

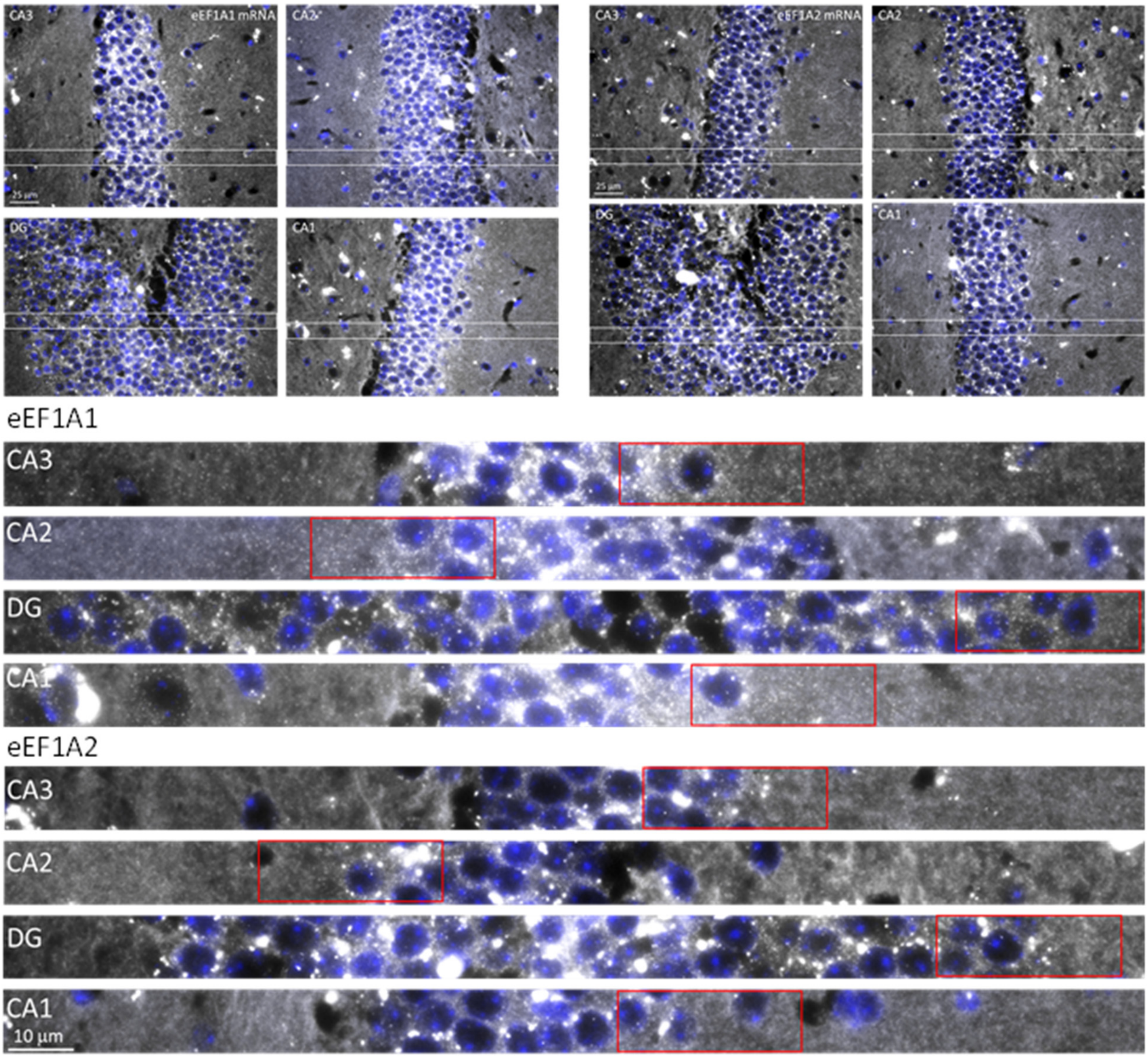
CA3
eEF1A1 mRNA
CA2
25 µm
DG
CA1
CA3
eEF1A2 mRNA
CA2
25 µm
DG
CA1
eEF1A1
CA3
CA2
DG
CA1
eEF1A2
CA3
CA2
DG
CA1
10 µm

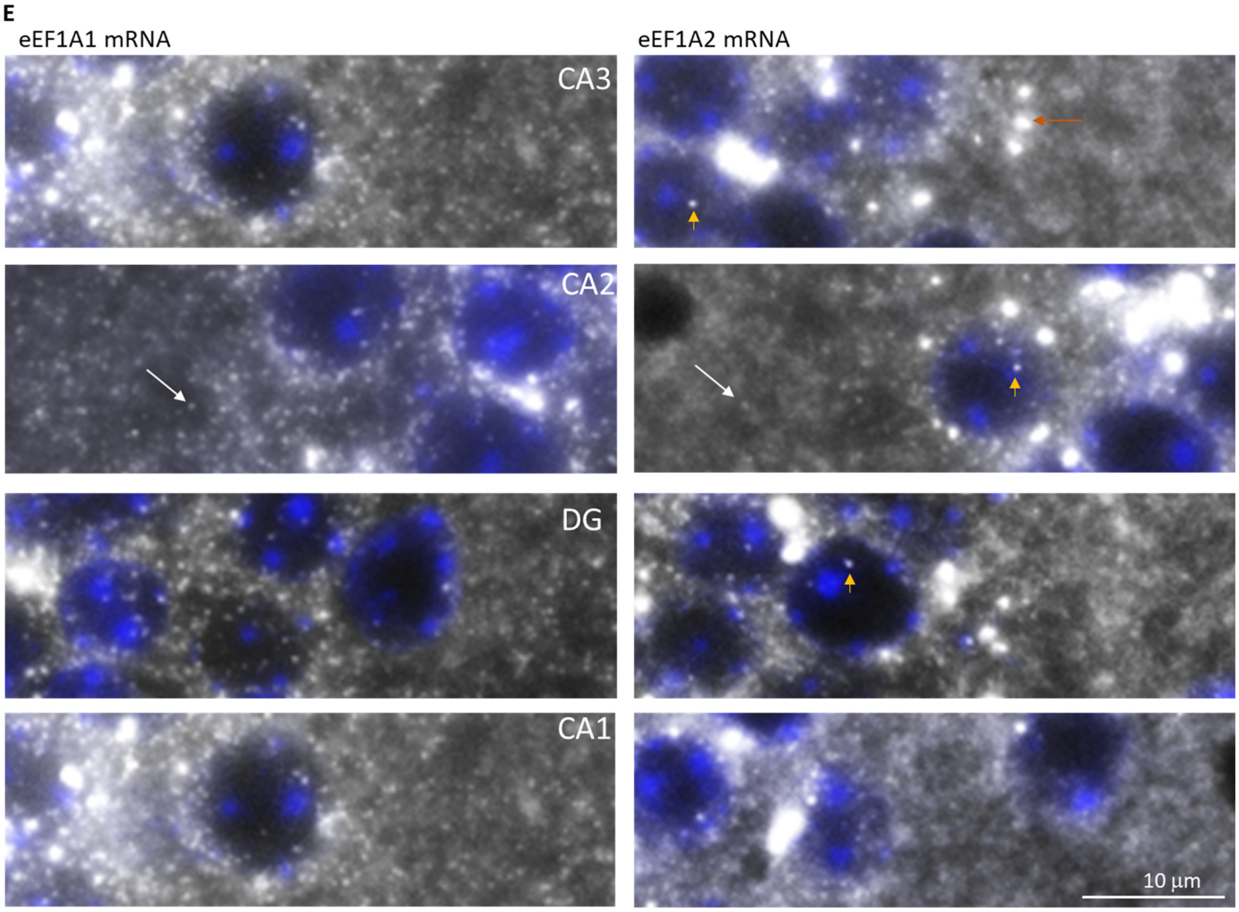

Figure S1. *eEF1A1* and *eEF1A2* mRNA localization in the dendrites through neurodevelopment

A-B. Density of mature *eEF1A1* (B) and *eEF1A2* (C) mRNAs per soma expressed as number of mRNAs per pixel of soma through neurodevelopment. **C-**. Tissue smFISH detects *eEF1A1* and *eEF1A2* mRNAs in the the hippocampus of 6-week-old mouse. Images were taken with a 60x objective, and the indicated cell area is magnified to show single mRNA molecules (white arrow points to a representative molecule) and transcription sites (yellow arrow points to a representative TS). **D-E**. Tissue smFISH detects *eEF1A1* and *eEF1A2* mRNAs in neurons of the hippocampus of 6-week-old mouse. Images were taken with a 60x objective, and the indicated area is magnified to show single mRNA molecules (white arrow points to a representative molecule) and transcription sites (yellow arrow points to a representative TS) in neurons. Blue is nucleus stained with DAPI. **D-E**. Tissue smFISH detects *eEF1A1* and *eEF1A2* mRNAs in neurons of the indicated regions of the hippocampus of 90-week-old mice. Images were taken with a 60x objective, and the indicated white rectangle is magnified to show single mRNA molecules across the tissue section in the soma and neurite areas (**D**). These sections are furthered magnified in **E** to show single mRNAs (white arrows), TSs (yellow arrows) and autofluorescence aggregates (orange arrows) that were more prevalent in 90 than 6-week-old mouse. Blue, nucleus stained with DAPI.

X. Appendix SA. Code information/ Program Layout

ARLIN (Analysis of RNA Localization In Neurons) is a program is composed of 6 modules, two of which are executable, part1.py and part2.py. Both Part 1 and Part 2 draw from ui.py and segmentation.py. **Fig. 1D** describes the purpose of each module and the module dependencies.

Part 1

Terminology:

A **print** is a 2D binary image for a specified cellular compartment, where there is a white pixel where the cellular exists and a black pixel everywhere else

A **skeleton** is a 2D binary image of a single white line representing the midline of a print for a dendrite

MAP2 is an immunofluorescence using an antibody that detects the Microtubule associate protein 2 (Map2) which localizes in dendrites

DAPI (4′,6-diamidino-2-phenylindole) is a blue-fluorescent DNA stain used to identify nuclei

Generating prints

Representative Image of prints is provided in **Figure 1D**. For dendrites, the program will look at each color used to annotate a dendrite in the annotation image and uses that color block as a mask for the 2D projection of the MAP2 Image. In other words, the program extracts the map2 signal colored over by the annotation, and it does so separately for each dendrite. This MAP2 signal is subsequently binarized using the Otsu threshold generated from the original unmasked 2D MAP2 Image. Thus, we obtain a binary image for each dendrite which is cleaned, smoothed, and then passed through an algorithm to account for inconsistent MAP2 signal.

Since synapses protrude out of dendrites, often the synapses are not contained by the Map2 stain, and thus also not contained by the dendrite prints. To account for this, when analyzing synapses, Part 1 of our program generates two prints for each dendrite, a regular one and another which is slightly dilated so that it contains all synaptic protrusions.

Because somas are easy to annotate accurately, when generating prints for somas, the program simply uses the masks generated from the annotations as a print. Thus, no binarizing is necessary, and only minimal cleaning is required to smooth the edges of the print.

For nuclei, the program will look at each color used to annotate a soma as a mask on the 2D projection of the DAPI channel. So, the program isolates the nuclei associated with each soma. The masked Image is then binarized using a scaled Otsu threshold of the 2D DAPI image. Qualitatively, we found that scaling the threshold down by 0.65 worked best. DAPI stains tend to be stronger around the perimeter of the nucleus and spotty in the center, so once a binary image of each nucleus is obtained, it is cleaned, by filling in holes and smoothing edges.

Algorithm for inconsistent signal

The MAP2 stain for dendrites varies in intensity even along the same dendrite. To obtain connected prints for each dendrite we merge fragments of the dendrite print with the following algorithm:

Input: 2D projection of a dendrite binarized by Otsu threshold

Algorithm:

- Save a copy of the input
- Obtain an over-dilated dendrite by dilating every pixel by a radius of 4 and then erode every pixel by a radius of 3 until all the white pixels in the Image are part of 1 connected component
- Skeletonize the over-dilated dendrite
- Dilate the skeleton by a 5-pixel radius
- Add the dilated skeleton to the copy of the original input and obtain the final print

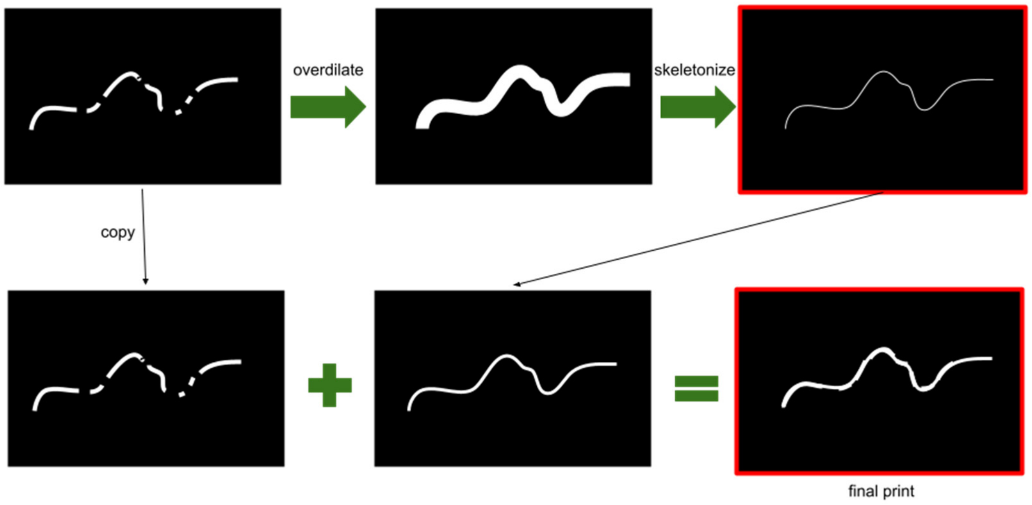

Figure S2. Schematic of the algorithm for inconsistent MAP2 signal

We choose to dilate by a radius of 4 pixels and erode by a radius of 3 pixel because it both smooths the edge of the Image while also having the net effect of dilating each pixel in the dendrite by a radius of 1. Also note that because the dendrite is over-dilated by expanding each pixel equally in all directions, the midline (ie skeleton) of the original input should not be greatly affected by the algorithm.

Trimming Skeletons

Initial skeletons are obtained using the skimage skeletonize function. However, sometimes these original skeletons have some small branches. To trim these branches, use the FilFinder library to extract the part of the skeleton corresponding to the longest path. Additionally, the initial skeletons on occasion contain small circle or line segments disjoint from the main skeleton. In these cases, our CleanBinaryImage function is applied, which keeps only the largest component of the skeleton image.

Cleaning Binary Images

Even though every dendrite print is passed through the algorithm for inconsistent signal the final prints may still have white blobs which are disjoint from the main print. This can happen when the saved copy of the original dendrite print captures a signal that is noise and not from the dendrite. So even though the algorithm for inconsistent signal dilates the print into 1 connected component, when the skeleton is added back to the saved copy, the extraneous white blobs are still present.

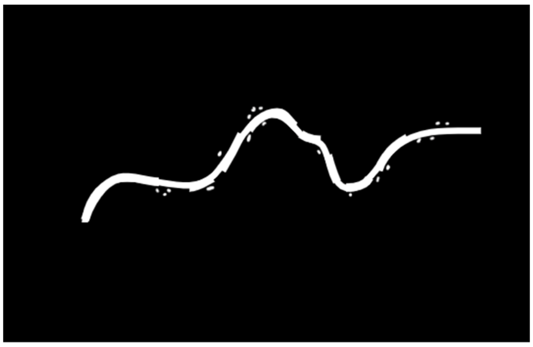

Figure S3. Example noisy signal spots surrounding print after applying the algorithm for inconsistent signal

This issue is accounted for with a function for cleaning binary images, named CleanBinaryImage. This function looks at every connected component in the print and deletes all components except for the one with the largest area. However, it first checks that the second-largest component has an area smaller than 20% of the area of the largest component. If the print does not pass this requirement, it is evidence that there is an abnormally large extraneous signal or that something else has gone wrong in the generation of the print. In this case, the program will not delete any small blobs from the print, will report the problem to the user, and will not add this dendrite to any of the outline files.

Note the program also contains a similar function for refining soma and nucleus prints, named refineSomaOrNucPrint which calls the Clean Binary Image function. Because DAPI stains tend to be spotty at center of the nucleus, in addition to deleting small white blobs the program also fills in holes

Saving Skeletons and Prints

All prints and skeletons are saved as black and white gif images in a folder called SkeletonsAndPrints, so that they can be accessed by the second part of the program.

FISHQuant

FISHQuant is a program that searches smFISH images for Gaussian distributions of signals to detect mRNAs (Mueller et al., 2013). Furthermore, the program can distinguish between multiple mRNA spots if their signals overlap, making it particularly useful for counting the number of mRNAs in transcription sites. We use version one of FISHQuant, which is written in Matlab, to find the coordinates of all the mRNA in each dendrite and soma.

Part 2

This part of the pipeline can be used to extract different information and statistics from the mRNA/synapse coordinates and dendrite morphology to provide insights for different biological questions.

Currently functionalities include finding:

1. Density of mRNAs within each dendrite and soma under different experimental conditions
2. Distribution of mRNAs along the dendrites based on how far they travel from the soma
3. Colocalization of different mRNA species based on how far each mRNA is from its closest mRNA from the other species
4. Colocalization of mRNAs with synapses based on how many mRNAs are within a threshold distance from each synapse

Input Extraction

We first parse the information in the text files generated by performing spot detect in FISHQuant which records the coordinates of every mRNA and synapse. Thus, we first extract all the coordinates and which dendrite or soma they came from. It should be noted that the output of FISHQuant's spot detection function flips the x and y coordinates. We account for this in our own program.

```
FISH-QUANT
File-version	3D_v1
RESULTS OF SPOT DETECTION PERFORMED ON 17-Aug-2021
COMMENT	Automated outline definition (batch or quick-save)
IMG_Raw	21-08-07_DIV14_MG132_HSPA8HSP110MAP2Tau_001_xy6_CY5.tif
IMG_Filtered	21-08-07_DIV14_MG132_HSPA8HSP110MAP2Tau_001_xy6_CY5_filtered_batch.tif
IMG_DAPI	21-08-07_DIV14_MG132_HSPA8HSP110MAP2Tau_001_xy6_DAPI.tif
IMG_TS_label
FILE_settings	_FQ_batch_settings_MATURE_210817.txt
PARAMETERS
Pix-XY	Pix-Z	RI	Ex	Em	NA	Type
107.5	300	1.35	547	583	1.4	widefield
CELL_START	Cell_1
X_POS	1146	1136	1135	1136	1142	1148	1152	1156	1160	1164	1167	1170	1174	1177	1182	1186	1191	1196	1200	1205
1208	1211	1215	1219	1223	1226	1230	1233	1237	1241	1246	1251	1255	1259	1263	1267	1272	1279	1286	1293	1299
1304	1309	1315	1321	1327	1334	1340	1349	1357	1362	1368	1377	1385	1386	1385	1384	1383	1381	1380	1381	1382
1384	1387	1391	1394	1397	1401	1406	1412	1422	1431	1430	1426	1422	1420	1416	1413	1410	1408	1407	1407	1409
1410	1411	1412	1412	1411	1408	1400	1392	1395	1391	1381	1371	1365	1359	1355	1351	1345	1336	1330	1325	1320
1313	1306	1299	1293	1289	1285	1281	1277	1272	1267	1262	1258	1255	1251	1248	1245	1241	1237	1233	1227	1223
1218	1216	1212	1208	1203	1199	1196	1193	1189	1186	1182	1177	1171	1163	1157
Y_POS	1632	1628	1618	1608	1598	1589	1579	1569	1559	1549	1539	1529	1519	1509	1499	1489	1479	1469	1459	1449
1439	1429	1419	1409	1399	1389	1379	1370	1360	1350	1340	1330	1320	1310	1300	1290	1280	1270	1260	1250	1240
1230	1220	1210	1200	1190	1180	1170	1160	1150	1140	1130	1120	1110	1100	1090	1080	1070	1060	1050	1040	1030
1020	1010	1000	990	980	970	960	950	946	954	964	974	984	994	1004	1014	1024	1034	1044	1054	1064
1074	1084	1094	1104	1114	1124	1134	1144	1154	1164	1171	1178	1188	1198	1208	1218	1226	1228	1238	1248	1258
1268	1278	1288	1298	1308	1318	1328	1338	1348	1358	1368	1378	1388	1398	1408	1418	1428	1438	1447	1457	1467
1477	1487	1497	1507	1517	1527	1537	1547	1557	1567	1577	1587	1597	1607	1617
Z_POS
CELL_END
SPOTS_START
Pos_Y	Pos_X	Pos_Z	AMP	BGD	RES	SigmaX	SigmaY	SigmaZ	Cent_Y	Cent_X	Cent_Z	MuY	MuX	MuZ	ITERY_det	Y_det	X_det	Z_det	Y_min
Y_max	X_min	X_max	Z_min	Z_max	INT_raw	INT_filt	SC_det	SC_det_norm	TH_det	TH_fit	IN_nuc
101690	152514	2908.29	1835.32	2167.74	1.27729e+07	145.371	145.371	469.374	212.892	217.42	935.074	209.894	186.569	1108.29	19	947	1420	10	945	949
1418	1422	7	13	4048	1568	384.017	0.70652	1	1	-1
101842	152880	5366.55	1144.3	1807.19	3.90548e+06	177.385	177.385	734.614	208.394	217.423	863.847	147.054	230.446	566.551	13	949	1423	20	947	951
1421	1425	17	23	3152	1083	211.998	0.390037	1	1	-1
102228	152291	4300.86	1503.02	1500.37	5.52946e+06	176.124	176.124	629.87	212.796	227.612	910.361	210.421	285.804	1000.86	13	952	1417	15	950	954
1415	1419	12	18	3124	1357	282.687	0.520092	1	1	-1
102307	152724	4466.42	1045.38	1932.88	4.18547e+06	120.941	120.941	446.139	210.313	213.853	911.432	182.49	181.256	866.418	13	953	1422	16	951	955
1420	1424	13	19	2972	919	172.12	0.316669	1	1	-1
102645	152300	5324.5	1358.95	1150.66	5.64583e+06	221.167	221.167	868.026	211.809	227.703	885.021	197.023	294.936	824.498	17	956	1417	19	954	958
1415	1419	16	22	3038	1399	243.953	0.448829	1	1	-1
102651	152757	5882.52	1303.38	1931.89	3.69842e+06	99.0517	99.0517	421.017	211.716	217.407	900.037	203.269	214.236	1082.52	12	956	1422	20	954	958
1420	1424	17	23	2973	1020	218.634	0.402246	1	1	-1
102758	153186	5077.13	1181.59	1826.66	2.81195e+06	162.434	162.434	455.275	223.315	214.826	901.768	310.716	213.25	877.132	9	956	1426	18	954	958
1424	1428	15	21	3065	990	196.358	0.361263	1	1	-1
102984	153732	3939.35	1490.67	2173.04	8.49488e+06	142.274	142.274	976.877	216.2	228.433	892.006	213.814	329.784	939.351	10	959	1430	14	957	961
1428	1432	11	17	3817	1353	369.906	0.680558	1	1	-1
102984	153697	3562.77	1717.4	1477.84	5.96186e+06	266.092	266.092	1111.04	203.885	221.875	978.115	106.338	294.218	1762.77	12	960	1430	10	958	962
1428	1432	7	13	3262	962	300.933	0.55366	1	1	-1
103283	153245	5912.66	1295.31	1878.27	1.31519e+07	199.973	199.973	583.651	209.4	209.633	926.882	190.392	165.103	1112.66	15	962	1427	20	960	964
1425	1429	17	23	3406	1346	354.786	0.65274	1	1	-1
103452	151888	3813.92	1331.32	952.534	2.81769e+06	162.15	162.15	742.932	222.873	236.048	884.348	251.644	313.366	813.919	13	963	1413	14	961	965
1411	1415	11	17	2367	1068	223.562	0.411313	1	1	-1
103710	152359	5755.58	1103.05	1296.57	2.49922e+06	154.334	154.334	677.837	210.296	220.527	863.1	187.068	246.673	655.579	14	966	1418	21	964	968
1416	1420	18	24	2573	1132	210.045	0.386443	1	1	-1
103850	151933	3016.45	1359.84	1269.04	4.57739e+06	149.311	149.311	596.772	216.332	222.509	903.197	219.989	250.038	916.452	11	967	1414	11	965	969
[illegible]
```

Figure S4. Example of output file from FISHQuant. The x and y coordinates of the mRNA or synapse signals are extracted for analysis.

Calculating Density

To calculate the area of a dendrite or soma, the program counts the number of pixels in the corresponding print generated from part 1. The area in pixels can then be converted to squared

nanometers (nm^2) based on the conversion factor specific to each microscope. We then can take the ratio of the number of mRNA in and the area of each dendrite/soma to obtain compartment-specific mRNA density.

Number	Channel	Number of mRNA	Area (sq. nanometer)	Density
3	CY3	36	2808168.75	0.0000128197424033011
3	CY5	36	2808168.75	0.0000128197424033011
4	CY3	36	2657937.5	0.0000135443365391398
4	CY5	36	2657937.5	0.0000135443365391398
6	CY3	36	2877506.25	0.0000125108329477999
6	CY5	36	2877506.25	0.0000125108329477999

Figure S5. Example of compartment-specific mRNA count, area (in pixels) and mRNA density.

Finding the Endpoints of Dendritic Skeleton

To find the two endpoints of the skeleton, the program looks at the neighbors of each white pixel in the skeleton. If a skeleton point is an endpoint, only one of its neighboring pixels will be white, while all other points in the skeleton will have two neighboring white pixels. Since the skeleton can have a curved structure, the neighboring points of a skeleton point can be anywhere inside a 3 by 3 grid centered at the skeleton point of interest. As a result, the program counts the number of white pixels in a 3 by 3 grid centered at each point in the skeleton. If there are 3 white pixels in the grid, the point of interest is considered to not be an endpoint. If there are only 2 white pixels, the skeleton point centered is an endpoint.

If the program finds less than 2 or more than 2 endpoints for a particular skeleton it is an indicator that the outline for this dendrite was not generated properly. In this case, the program will not include the dendrite associated with the skeleton in the statistical analysis.

Determine the Soma Endpoint

Once two endpoints of the skeleton are found, the program aims to determine which of the two endpoints belongs to the soma end. Since dendritic annotation does not keep track of the soma end, the program needs to determine it using available information. With thousands of images from previous smFISH experiments, it has been observed that the soma end of each dendrite almost always has more mRNAs than the distal end. Biologically, this observation can be explained by the fact that the transportation of mRNAs out of the soma is limited. Thus, we assume that the end of the dendrite with more mRNA is the end closer to the soma.

Distance of mRNA to soma

To find how far the mRNAs are from the soma, the program first projects each mRNA to the closest pixel on the skeleton. Then it counts the number of pixels along the skeleton from the soma endpoint to the projected mRNA. As we count pixels along the skeleton, we keep track if an adjacent or

diagonal pixel step was taken, so that we can calculate the precise distance of each step (sqrt(2) for a diagonal step and 1, for an adjacent step). Note that the distance along the skeleton from an mRNA to an endpoint is more accurate than the Euclidean distance because it considers the curved morphology of the dendrite. Since the skeleton is the center axis of the dendrite, the skeleton and dendrite share the same curvature. The calculated distances are then binned into groups of 25 micrometers before being written to an excel file.

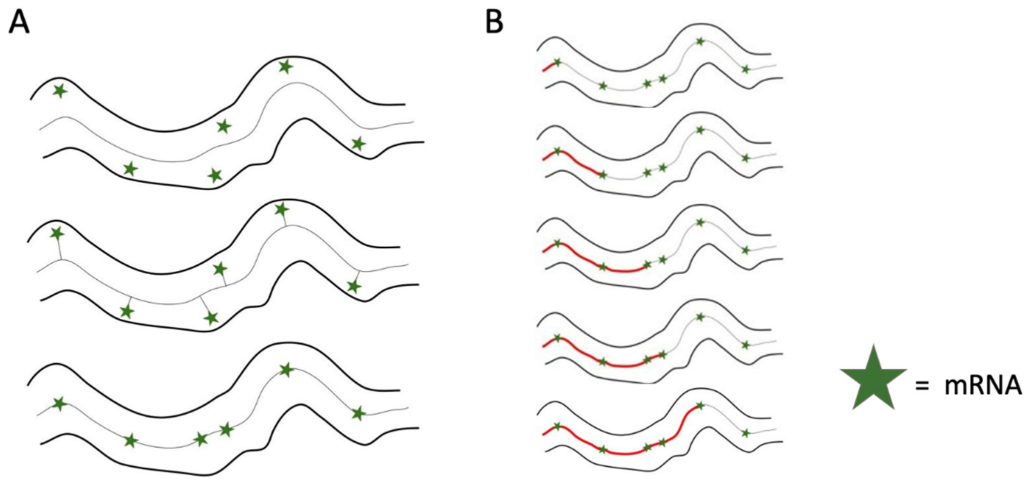

Figure S6. A) Diagram of mapping mRNA spot coordinates to the skeleton B) Measuring distance of mRNA to soma by counting pixels along the skeleton

Dendrite Num	Channel	0-25 (um)	25-50 (um)	50-75 (um)	75-100 (um)	100-125 (um)	125-150 (um)	>= 150 (um)
1	Cy3	89	35	33	32	35	22	0
1	Cy5	47	9	8	13	10	6	0
2	Cy3	52	36	42	2	0	0	0
2	Cy5	45	16	12	0	0	0	0
3	Cy3	67	40	26	48	2	0	0
3	Cy5	61	25	13	25	0	0	0
4	Cy3	110	0	0	0	0	0	0
4	Cy5	68	0	0	0	0	0	0
5	Cy3	90	64	68	71	55	0	0
5	Cy5	51	40	26	35	32	0	0
6	Cy3	74	43	34	30	31	16	0
6	Cy5	38	14	13	4	9	3	0
7	Cy3	59	36	35	0	0	0	0
7	Cy5	52	20	10	0	0	0	0
8	Cy3	181	0	0	0	0	0	0
8	Cy5	96	0	0	0	0	0	0
9	Cy3	101	69	49	30	4	0	0
9	Cy5	73	26	12	5	1	0	0
[illegible]	[illegible]	[illegible]	[illegible]	[illegible]	[illegible]	[illegible]	[illegible]	[illegible]

Figure S7. Example of mRNA distribution based on the distance to soma along with the skeleton; each dendrite is assigned a unique number

mRNA Colocalization Statistics

Our motivation for calculating colocalization statistics for two kinds of mRNA is to see if the mRNAs are moving together down the dendrites in granules. The program will loop through every mRNA of species A and find the distance to the closest mRNA of species B. The same calculation can be done the other way around, finding the distance between each mRNA of species B to the closest mRNA of species A. Additionally, the program calculates self-colocalization, the distance

between an mRNA of species A to another mRNA of species A (not including itself). To have a computational control, the same colocalization statistics were calculated with simulated mRNA coordinates. The simulation process is described below in the section "Simulation as Control." Nearest distances are then binned into user-defined groups before being written to an excel file.

Distance (nm)	Cy3 to closest Cy5	Cy5 to closest Cy3	Cy3 to closest Cy3	Cy5 to closest Cy5	Sim-Cy3 to closest Sim-Cy5	Sim-Cy5 to closest Sim-Cy3
0-75	487	489	94	109	0	0
75-150	1172	1190	387	359	0	0
150-225	1216	1198	710	550	0	0
225-300	1203	1113	988	671	0	0
300-375	792	655	1183	628	0	0
375-450	516	308	1191	643	0	0
450-525	448	187	1108	587	0	0
525-600	385	107	1087	518	0	0
600-inf	2863	277	2910	2168	9082	5524

Figure S8. Example of mRNA colocalization statistics: mRNAs are binned based on Euclidean distance to the closest mRNA of the other species of interest

Synapse Statistics

We seek to understand how often synapses are being served by mRNA. We assume that mRNAs localizing near a synapse are servicing that synapse. We take advantage of the fact that PSD95 staining for a single synapse looks like a single mRNA spot and detects the position of synapses using FISHquant. When part2.py is run, the program will ask the user to define a colocalization threshold which will be used to determine whether a certain mRNA is localized at a synapse. Then the program loops through every synapse coordinate of each dendrite and counts the number of mRNAs that are within a radius of the threshold distance. We record the number of synapses with X localized mRNA, for X equal to 0, 1, 2, 3, etc. As with the mRNA colocalization functionality, a computation control is required to interpret the statistical significance of the degree of synaptic localization. We generate fake mRNA coordinates and again calculate the number of synapses being served by X fake mRNA.

Num of mRNA	Real Cy3	Sim Cy3	Num of mRNA	Real Cy5	Sim Cy5
0	1724	2164.61	0	2148	2394
1	525	109.73	1	358	140.48
2	150	78.5	2	100	60.24
3	32	46.14	3	16	23.12
4	5	23.15	4	6	7.87
5	1	10.21	5	0	2.4
6	1	3.84	6	1	0.64
7	0	1.39	7	0	0.2
8	0	0.32	8	0	0.04
9	0	0.1	9	0	0.01
10	0	0			
11	0	0			
12	0	0.01			

Figure S9. Example of synapse colocalization statistics: synapses are binned based on how many real or simulated mRNAs of interest are within its threshold radius

Simulation as Control

It is necessary to verify that the observed colocalization patterns (between mRNA and synapses) are due to meaningful biological mechanisms (e.g., active transport of mRNA to a synapse, co-transportation of different mRNA species) rather than restricted space and randomness. To do this, the program picks N fake mRNA sub-pixel coordinates uniformly random from each print, where N is the number of real mRNA in that dendrite. The program then uses these fake mRNA coordinates to calculate statistics in the same way it would with real mRNA coordinates. This simulation is repeated 100 times (50 times in the older version of the program), and averages of each statistic are taken. The simulation serves as computation control because if the simulated mRNAs show the same degree of colocalization as the real mRNAs, then colocalization patterns are likely due to having many mRNAs crammed into a restricted space. However, if the real mRNAs show strong colocalization while simulated mRNAs do not, it suggests the existence of an underlying biological mechanism supporting mRNA localization.

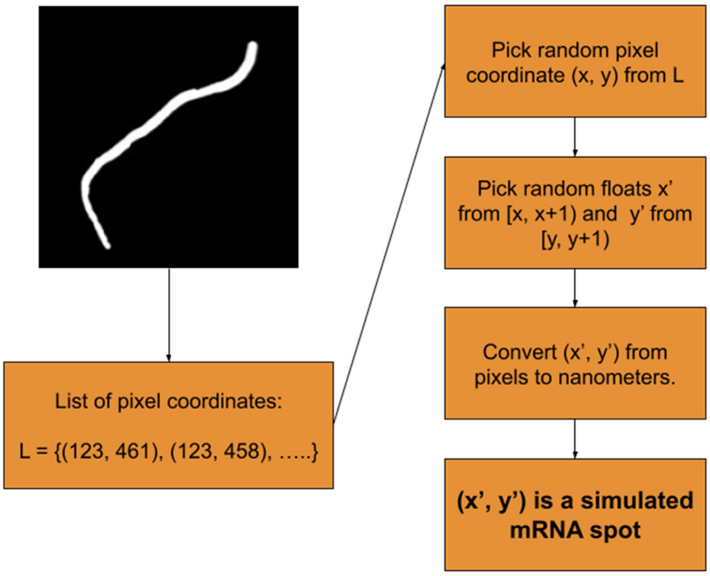

Figure S10. Steps used to uniformly randomly sample sub-pixel coordinates from dendrite print to use as simulated mRNA spots

Writing to Excel Files

The results of all distribution and colocalization analyses are organized into tables and then saved as excel files. The tabular results can then be used to generate plots for visualization and examination.

Disclaimer: The program layout below is for the newer modularized version of our code. An older version of the code was used to collect the data presented in the paper. Both versions used the same fundamental algorithms, but the older version was organized so that each functionality of part2 was in its own executable script.

References

Mueller, F., Senecal, A., Tantale, K. *et al.* FISH-quant: automatic counting of transcripts in 3D FISH images. *Nat Methods* 10, 277–278 (2013). https://doi.org/10.1038/nmeth.2406

XI. Appendix SB. User Manual

Part 1

1. Setting up your folder of images

We recommend having a specific folder containing all the materials for a specific experiment. Within this experiment folder you should have a subfolder containing all your microscopy images in ".tif" file format.

2. Naming your images

It is important that your images are named properly so that the program can read in all your images.

All images should be names as follows:

***ExperimentName*_(DIV or div)##_*treatment*_*listOfStains*_00#(xy or XY)##_*channel*.gif**

Where the DIV ## is the age of the neurons. The treatment is the experimental condition. The numbers before and after xy/XY specify the Field of View (FOV), and the channel is the code specifying the microscope channel used for acquiring that Image. You should have one channel for Map2, one for DAPI, a few channels for various mRNAs, and an optional channel for a synaptic stain like PSD95.

Below is an example of the folder setup and image names for a small experiment with DIV 1 and DIV 2, and a treatment called "cta".

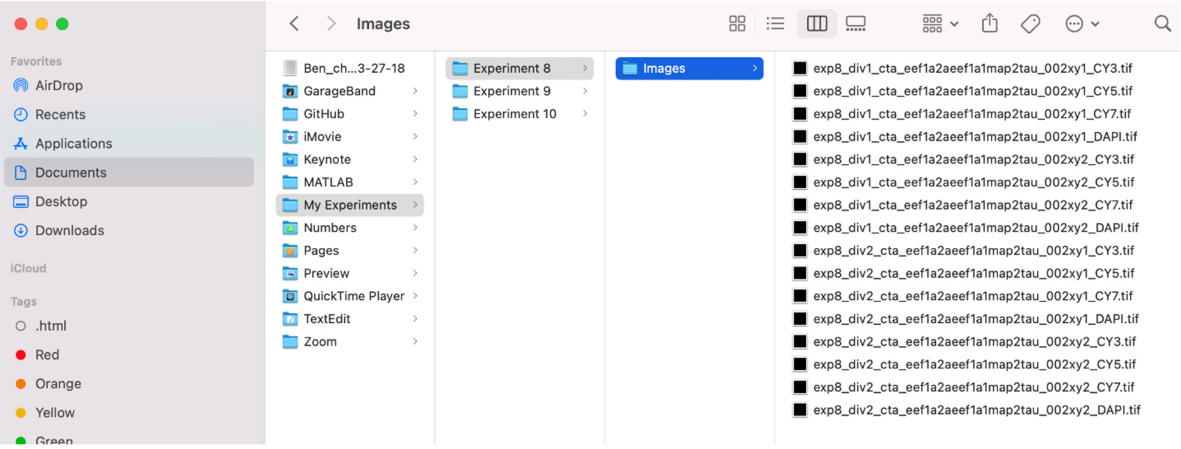

<u>**Note S1:**</u> If you are doing other types of experiments such that you don't record the DIV number or treatment, you can either add a dummy word there or change the regex expression that the code uses to read images. To do this, alter the regex expression in part1.py and part2.py and change the MaxImg object definition in ui.py.

3. Obtaining 2D projections

The first step is to use ImageJ (aka Fiji) to obtain 2D max projections of your ".tif" images. To do this: 1. Open each Image in Fiji and go to Image > Stacks > Z Project. 2. In the pop-up window, make sure the projection type is "Max Intensity". 3. Save your Image as a gif into your folder of images.

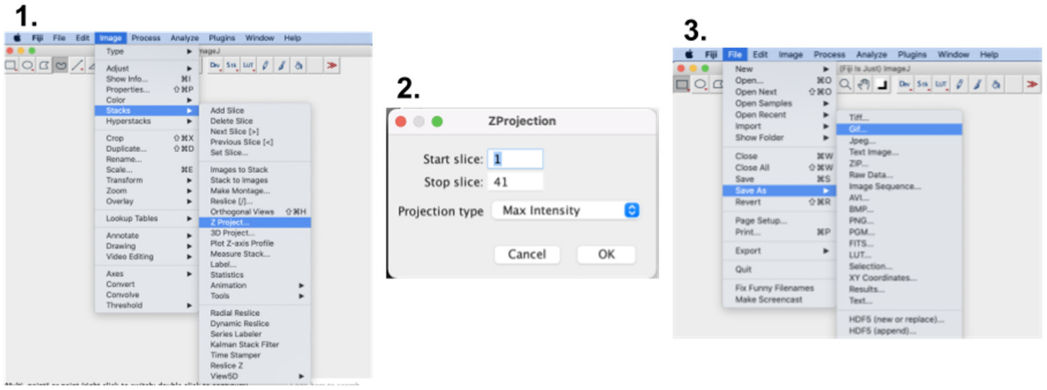

<u>Note S2:</u> If you are only analyzing somas and dendrites, you will only need max projections of your Map2 (dendrite stain) images. If you are also analyzing nuclei, you will also need to get max projections of your DAPI (nuclei stain) images.

4. Making annotations

To annotate dendrite or somas, open the Map2 max projection .gif in a simple image editing software, for example, Preview on a Mac or Paint on a PC.

Color over up to 9 dendrites or somas of interest **using the following colors in the following order**

1. Red [RBG: (255, 0, 0), hex: #ff0000]
2. Green [RBG: (0, 255, 0), hex: #00ff00]
3. Blue [RBG: (0, 0, 255), hex: #0000ff]
4. Orange [RBG: (240, 134, 51), hex: #f08633]
5. Yellow [RBG: (255, 255, 0), hex: #ffff00]
6. Purple [RBG: (143, 57, 182), hex: #8f39b6]
7. Teal [RBG: (130, 210, 208), hex: #82d2d0]
8. Mint [RBG: (214, 253, 208), hex: #d6fdd0]
9. Salmon [RBG: (255, 128, 102), hex: #ff8066]

You must be sure to use exactly these colors with these RBG values. If you are using Preview, you can change the precise color of your pen by clicking on the color, clicking on "Show colors..." and specifying the Hex code, or sliding the RBG values.

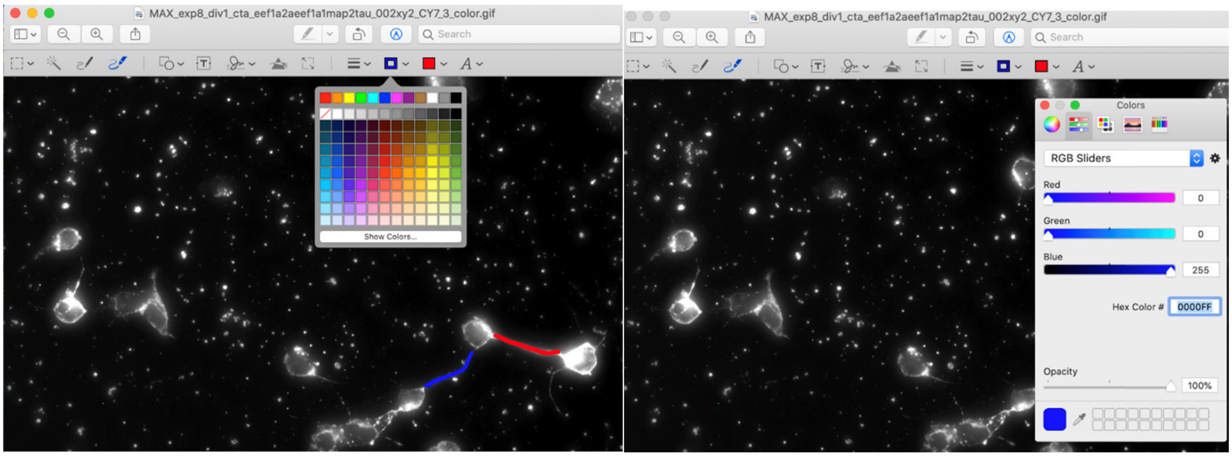

<u>Note S3</u>: We have found that sometimes when saving gif images from Paint on PC's, the colors of the annotations can change. This affects the ability of the program to detect the annotations. If you annotate Image on a PC, we recommend that you open images of the annotations you have made and record the RBG values of the color that the annotations were saved as. Then open part1.py and on lines 11-20 edit the RBG values of each color accordingly.

```
9   def main():
10      #####Set colors######
11      colors = {"red": [255,0,0],
12              "green": [0,255,0],
13              "blue" : [0,0,255],
14              "orange" : [240, 134, 51],
15              "yellow" : [255, 255, 0],
16              "purple" : [143, 57, 182],
17              "teal" : [130, 210, 204],
18              "mint" : [214, 253, 208],
19              "salmon": [255, 128, 102]
20              }
```

4.a If analyzing dendrites:

- Make a copy of each 2D Map2 gif and open it in your Image annotating software
- Color over up to 9 dendrites, using the colors in order as listed above
- Prints for dendrites are generated by extracting the signal behind your annotations, so it is best to cover the dendrites of interest completely
- Save the gif
- Rename the file by adding the tag "dendAnnot_N" before "gif", where N is the number of dendrites you colored in that Image

4.b If analyzing somas/nuclei:

- Make a copy of each 2D Map2 gif and open it in your Image annotating software
- Color over up to 9 somas, using the colors in order as listed above

- Soma prints are generated by taking the area of the annotations, so it is more critical that soma annotations are accurate and do not extend past the edge of the soma
- Save the gif
- Rename the file by adding the tag "somAnnot_N" before "gif" where N is the number of somas you colored in that Image

4.c Tips for Annotating

- Try to avoid having your dendritic annotations touch the border of the Image as it can distort the skeleton of dendrites
- Before annotating a soma, open the DAPI max projection to make sure the nucleus for that soma is clearly visible
- Avoid annotating cellular compartments that overlap significantly with other dendrites or somas because FISHQuant will overcount the number of mRNAs in that cellular compartment
- Careful annotations lead to better prints and skeletons

Below is an example of how your 2D max projections and annotations should be named.

```
MAX_exp8_div1_cta_eef1a2aeef1a1map2tau_002xy1_CY7_2_dendAnnot.gif
MAX_exp8_div1_cta_eef1a2aeef1a1map2tau_002xy1_CY7_3_somAnnot.gif
MAX_exp8_div1_cta_eef1a2aeef1a1map2tau_002xy1_CY7.gif
MAX_exp8_div1_cta_eef1a2aeef1a1map2tau_002xy1_DAPI.gif
MAX_exp8_div1_cta_eef1a2aeef1a1map2tau_002xy2_CY7_1_dendAnnot.gif
MAX_exp8_div1_cta_eef1a2aeef1a1map2tau_002xy2_CY7_5_somAnnot.gif
MAX_exp8_div1_cta_eef1a2aeef1a1map2tau_002xy2_CY7.gif
MAX_exp8_div1_cta_eef1a2aeef1a1map2tau_002xy2_DAPI.gif
MAX_exp8_div2_cta_eef1a2aeef1a1map2tau_002xy1_CY7_3_dendAnnot.gif
MAX_exp8_div2_cta_eef1a2aeef1a1map2tau_002xy1_CY7_3_somAnnot.gif
MAX_exp8_div2_cta_eef1a2aeef1a1map2tau_002xy1_CY7.gif
MAX_exp8_div2_cta_eef1a2aeef1a1map2tau_002xy1_DAPI.gif
MAX_exp8_div2_cta_eef1a2aeef1a1map2tau_002xy2_CY7_1_dendAnnot.gif
MAX_exp8_div2_cta_eef1a2aeef1a1map2tau_002xy2_CY7_6_somAnnot.gif
MAX_exp8_div2_cta_eef1a2aeef1a1map2tau_002xy2_CY7.gif
MAX_exp8_div2_cta_eef1a2aeef1a1map2tau_002xy2_DAPI.gif
```

5. Now you are ready to run part1.py!

After running part1.py, your folder of images should have a new folder containing the text file outlines for dendrite and somas specific to each mRNA channel. You should also have a folder called **"SkeletonsAndPrints,"** which will contain all the gif images of the skeletons and prints for every annotated compartment.

At this point, your folder of images should look like the following:

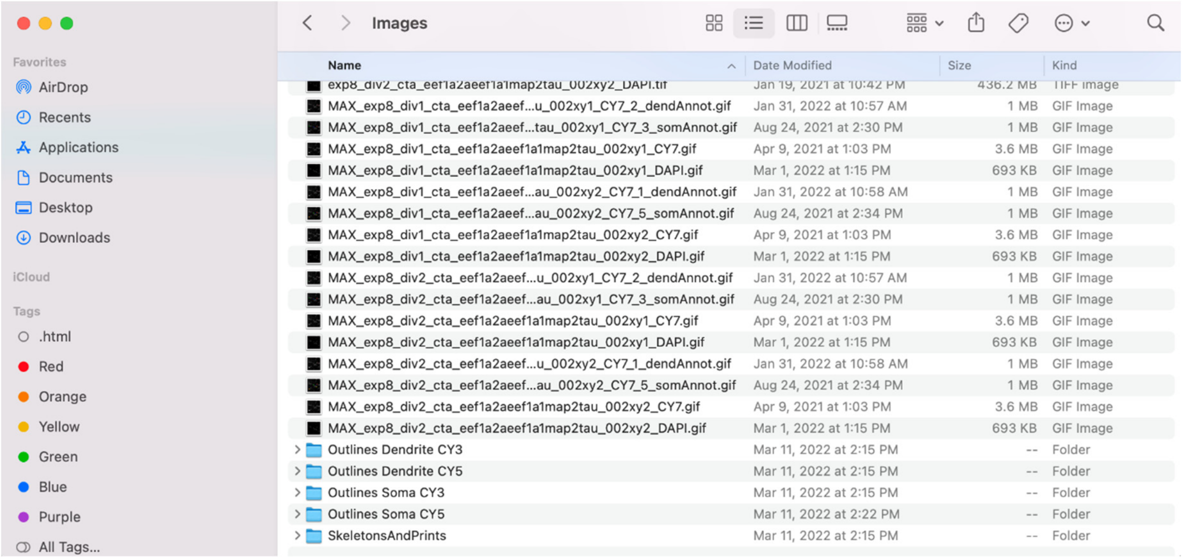

Within the **SkeletonsAndPrints** folder, each print/skeleton for a compartment is labeled with a unique **"segmentation number."** This segmentation number is recorded as the cell number in the text file outlines. The segmentation number for a soma is the same segmentation number as its corresponding nucleus.

<u>**Note S4:**</u> If an annotation was done with the wrong color, if a soma had no clear nucleus in the DAPI image, or if there was any other technical issue with generating a print for a compartment, the print will not be saved as a gif. In this case, we skip this compartment in the outline text files, and we also skip a segmentation number.

The matching segmentation numbers for a soma and its corresponding nuclei are circled below

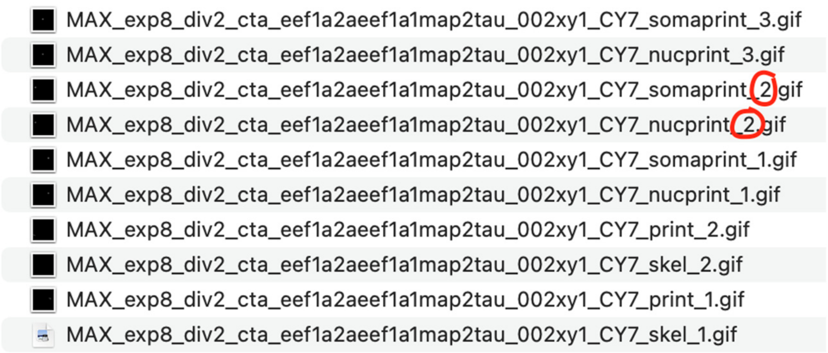

Now you should make new folders in your folder of images so that you have a place to save your result files from FISHQuant. You should name your folders **"Results Dendrite/Soma *channel*"** for each mRNA channel (and synaptic channel) you are using.

Below is an example of how to name your result folders

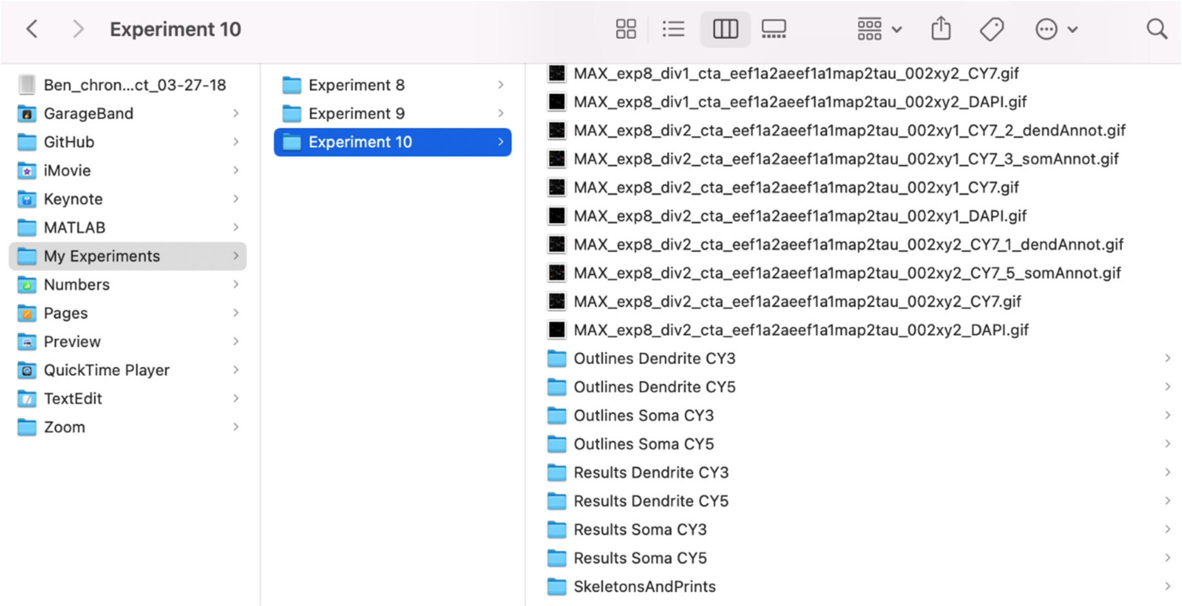

FISHQuant

You are now ready to run FISHQuant!

When you run FISHQuant, you can set the root folders with batch processing. Below is an example of how you would set the root folder for analyzing Dendrites for the CY3 channels...

- Root folder for images: "Images"
- Root folder for outlines: "Outlines Dendrites CY3"
- Root folder for results: "Results Dendrite CY3"

If you would like to do synaptic localization analysis in Part 2, you will need to also use FISHQuant to find the coordinates of every synapse. You can do this by treating your synapse stain the same as you would an mRNA stain.

For more information about using FISHQuant, reference this article (https://www.nature.com/articles/nmeth.2406)

Part 2

When you run part2.py the command prompt will ask you which of the following functionalities you would like to use:

1. *Density Analysis*
   - Input: None
   - Output: Excel file called SomaDensities.xlsx/DendriteDensisties.xlsx
   - Statistics: The density of mRNA per square nanometer in each annotated cellular compartment
   - Example:

Number	Channel	Number of mRNA	Area (sq. nanometer)	Density
1	Cy3	254	14087068.75	0.000018030720550008
1	Cy5	96	14087068.75	0.0000068147605228376
2	Cy3	132	6309712.5	0.0000209201290867056
2	Cy5	73	6309712.5	0.0000115694653282539
3	Cy3	183	8956093.75	0.0000204330152305518
3	Cy5	124	8956093.75	0.0000138453217955652
4	Cy3	110	5639450	0.0000195054482263341
4	Cy5	68	5639450	0.0000120579134490066
5	Cy3	348	11845156.25	0.0000293790974686383
5	Cy5	184	11845156.25	0.0000155337756730731
6	Cy3	242	13347468.75	0.0000181307785418115
6	Cy5	92	13347468.75	0.0000068926926687878

2. *Distribution Analysis*
   - Input: None
   - Output: Excel file called DistrAnalysis.xlsx
   - Statistics: The number of mRNA at 0-25, 25-50, 50-75, 75-100, 100-125, 125-150, >150 nm away from the soma for each annotated dendrite. Distances are measured along the skeleton for each dendrite
   - Example:

Dendrite Num	Channel	0-25 (um)	25-50 (um)	50-75 (um)	75-100 (um)	100-125 (um)	125-150 (um)	>= 150 (um)
1	Cy3	89	35	33	32	35	22	0
1	Cy5	47	9	8	13	10	6	0
2	Cy3	52	36	42	2	0	0	0
2	Cy5	45	16	12	0	0	0	0
3	Cy3	67	40	26	48	2	0	0
3	Cy5	61	25	13	25	0	0	0
4	Cy3	110	0	0	0	0	0	0
4	Cy5	68	0	0	0	0	0	0
5	Cy3	90	64	68	71	55	0	0
5	Cy5	51	40	26	35	32	0	0
6	Cy3	74	43	34	30	31	16	0
6	Cy5	38	14	13	4	9	3	0
7	Cy3	59	36	35	0	0	0	0
7	Cy5	52	20	10	0	0	0	0
8	Cy3	181	0	0	0	0	0	0
8	Cy5	96	0	0	0	0	0	0
9	Cy3	101	69	49	30	4	0	0
9	Cy5	73	26	12	5	1	0	0
10	Cy3	67	59	57	6	0	0	0
10	Cy5	56	31	25	4	0	0	0

3. *Colocalization Analysis*
   - Input: 2 channels of mRNA that are to be analyzed, max recorded distance, desired increment to record distances in the excel table
   - Output: Excel file called ColocAnalysis.xlsx
   - Statistics: In each dendrite, for each mRNA of type A we record the minimum distance to another mRNA of type B, and for each mRNA of type B we record the minimum distance to an mRNA of type A. This is also done for distance from type A to mRNA themselves and distance from type B mRNA to themselves. These distances are binned based on the desired increment and reported in the excel file. To provide a computational

control, we randomly simulate the coordinates of both type A and type B mRNA 100 times and report the average statistics using these simulated coordinates in the last two columns of the excel file.

- Example:

Distance (nm)	Cy3 to closest Cy5	Cy5 to closest Cy3	Cy3 to closest Cy3	Cy5 to closest Cy5	Sim-Cy3 to closest Sim-Cy5	Sim-Cy5 to closest Sim-Cy3
0-75	487	489	94	109	0	0
75-150	1172	1190	387	359	0	0
150-225	1216	1198	710	550	0	0
225-300	1203	1113	988	671	0	0
300-375	792	655	1183	628	0	0
375-450	516	308	1191	643	0	0
450-525	448	187	1108	587	0	0
525-600	385	107	1087	518	0	0
600-inf	2863	277	2910	2168	9082	5524

4. *Synapse Localization Analysis*
   - Input: Name of the synapse channel, threshold distance to consider mRNA localized at a synapse.
   - Output: Excel file called SynapseAnalysis.xlsx
   - Statistics: For every annotated dendrite and every synapse, we calculate the number of mRNA with the threshold distance to the synapse. We sum over all dendrites and record the number of synapses with X mRNAs localized at them, where X is 1, 2, 3, etc. To provide a computational control, we randomly simulate the coordinates of each type of mRNA 100 times and report the average statistics using these simulated coordinates in the columns titled "Sim *mRNA Channel*."
   - Example:

Num of mRNA	Real Cy3	Sim Cy3	Num of mRNA	Real Cy5	Sim Cy5
0	1724	2164.61	0	2148	2394
1	525	109.73	1	358	140.48
2	150	78.5	2	100	60.24
3	32	46.14	3	16	23.12
4	5	23.15	4	6	7.87
5	1	10.21	5	0	2.4
6	1	3.84	6	1	0.64
7	0	1.39	7	0	0.2
8	0	0.32	8	0	0.04
9	0	0.1	9	0	0.01
10	0	0			
11	0	0			
12	0	0.01			

Note S6: Part 2 will calculate the localization statistics separately for dendrites/somas of different DIV #s and treatments. Each excel file will have a sheet for each DIV treatment combination used in the experiment.
